# A Cell-Based Potency Assay for Determining the Relative Potency of Botulinum Neurotoxin A Preparations Using Manual and Semi-Automated Procedures

**DOI:** 10.3390/toxins18010045

**Published:** 2026-01-15

**Authors:** F. Mark Dunning, Sara Hendrickson, Serena Wolfe, Dan Harding, Theresa Geurs, Timothy M. Piazza, Thomas A. Little, Ward C. Tucker

**Affiliations:** 1 BioSentinel Inc., 505 S. Rosa Road, Madison, WI 53719, USA; mdunning@biosentinelpharma.com (F.M.D.); shendrickson@biosentinelpharma.com (S.H.); swolfe@biosentinelpharma.com (S.W.); tgeurs@biosentinelpharma.com (T.G.); tpiazza@biosentinelpharma.com (T.M.P.); 2 BioAssay Sciences, Highland, UT 84003, USA; daniel.harding@bioassaysciences.com (D.H.); drlittle@bioassaysciences.com (T.A.L.)

**Keywords:** botulinum neurotoxin, cell-based potency assay, relative potency, parallel line analysis, laboratory automation

## Abstract

Cell-based potency assays (CBPAs) are required for the potency testing and commercial release of botulinum neurotoxin (BoNT)-based drug products. These CBPAs must account for the toxin’s biological activities while meeting regulatory guidelines for precision and accuracy. Here, studies describe the characterization and qualification of the BoSapient CBPA and demonstrate that it is fit for use as a relative potency assay for BoNT/A-containing samples. The CBPA is operated in a 96-well plate format and relies upon the fluorescence emissions of a reporter that directly responds to BoNT/A activity. The BoSapient cell line expresses the BoNT/A-receptors SV2 and complex gangliosides, is responsive only to intact BoNT/A, and can robustly detect picomolar and sub-picomolar BoNT/A quantities, making the CBPA appropriate for quantifying BoNT/A-based drug products. The cell line was passaged 30 times without significant loss of reporter expression or BoNT/A sensitivity. Manual and semi-automated CBPA methods were developed and qualified according to regulatory guidelines and shown to have low bias (<4% from expected) and high precision (standard deviation < 8) across all test concentrations. Furthermore, the semi-automated method using the CBPA is demonstrated to improve intermediate precision by 39% compared to the manual method, while reducing operator dependency during method execution.

## 1. Introduction

Botulinum neurotoxins (BoNTs) are some of the most lethal substances known and are the causative agents of the disease botulism [1]. Produced by bacteria of the genus *Clostridium*, BoNTs are zinc-dependent endopeptidases that inhibit neurotransmitter release by selectively binding neurons, entering the neurons through vesicular endocytosis, and translocating into the cytosol [2,3]. BoNT’s endopeptidase activity then cleaves soluble *N*-ethylmaleimide-sensitive factor attachment protein receptors (SNAREs), which compromise the cellular machinery required for synaptic vesicle fusion and neurotransmitter release. BoNTs are di-chain molecules, each composed of a heavy chain (Hc) that is responsible for receptor binding and membrane translocation of the light chain (Lc). The Lc is responsible for substrate recognition and cleavage. There are at least seven BoNT serotypes, labeled A–G, which differ in their cell receptor and SNARE substrate specificity, as well as their toxicity profiles. Exposure to BoNTs can result in flaccid paralysis, respiratory failure, and death in both humans and animals [4,5,6,7].

BoNTs are highly specific for cellular receptors expressed by neurons, particularly neurons of the neuromuscular junction [8,9,10,11]. Due to their ability to inhibit neurotransmitter release at the neuromuscular junction, resulting in muscle paralysis, BoNTs are widely used in cosmetic and pharmaceutical applications where partial muscle paralysis at localized injection sites can provide smoothing of the skin or relief from a number of disorders and diseases [5,12]. Drug products containing BoNT serotype A (BoNT/A) and B (BoNT/B) are FDA- and EMA-approved for treating glabellar lines, strabismus, cervical dystonia, blepharospasm, cranial nerve VII disorders, chronic migraines, and primary axillary hyperhidrosis. Dozens of “off-label” BoNT clinical applications are also documented [13,14,15,16,17]. The therapeutic utility of BoNTs is expected to increase in the coming years as more pharmaceutical companies develop BoNT-based drug products, including products that contain modified BoNTs or new formulations with altered selectivity, duration, or application methods [18,19,20].

Commercial release of a BoNT-based drug product requires a means to determine drug product potency to ensure manufacturing consistency and patient safety. An acceptable potency testing method requires an assay that encompasses all of BoNT’s cellular activities: cell receptor binding, membrane translocation, and proteolytic activity [12,21,22]. The mouse-based potency assay (MBPA) is, historically, the standard method for testing BoNT-containing samples including drug products [23,24,25,26,27]. The MBPA provides excellent sensitivity (LOD < 50 pg) and reports botulism activity in the form of death following the classical signs of botulism (e.g., labored breathing, pinched waist, and ruffled coat). The MBPA, however, is also characterized by a narrow linear range, low precision, high assay failure rates, and animal welfare concerns [25,26,28,29]. A single testing event of a quantitative MBPA method can require > 100 mice that are kept in specialized housing and injected by highly trained personnel. For these reasons, drug product manufacturers and regulatory agencies have sought alternatives to the MBPA that reduce or eliminate the use of animals while retaining the ability to assess all BoNT’s cellular activities.

Cell-based potency assays (CBPAs) offer an attractive alternative to the MBPA as they do not require animals and, upon successful development, can offer higher precision and accuracy with lower failure rates than animal testing. When BoNT-containing samples are applied to an appropriate CBPA, BoNT binds to and is endocytosed into the cells, after which the Lc is translocated into the cytosol. The Lc then cleaves its SNARE substrate (SNAP-25 for BoNT/A). The CBPA may report toxin action either by direct detection of substrate cleavage [30,31], by identification of cleavage products [32,33,34,35,36,37], or by measured reduction in secreted cellular products (e.g., neurotransmitters, hormones, or peptides) [38,39,40]. CBPAs are typically executed as relative potency assays in which the response of a test sample in the CBPA is compared to the response of a reference sample [31,32].

Successful development of a CBPA for BoNT-containing drug products and substances has four basic requirements. First, the parental cell line from which the CBPA is developed must express the necessary receptors and pathways to replicate BoNT’s neuronal mechanisms of action. Second, a reliable means to detect BoNT activity must be developed. Third, assay sensitivity must be appropriate for the sample being tested. For example, a vial of 100 U BOTOX (Allergan, Irvine, CA, USA) contains ~5 ng of BoNT/A. A 1 mL suspension of the drug product would yield a concentration of ~5 ng/mL or ~5.6 pM (assuming the active ingredient is the 900 kDa BoNT/A complex [41]). Thus, for any CBPA to be useful as a relative potency assay for BoNT-based drug products, the CBPA must be able to reliably detect BoNT concentrations in the low to sub-picomolar range. Finally, the resulting assay must be able to precisely, accurately, and robustly determine the relative potency of BoNT-containing samples over time.

CBPAs for BoNT detection are described in the literature, including assays that are currently regulatory-approved for determining BoNT-based drug product potency [31,32]. These CBPAs detect BoNT activity in cultured primary and stem-cell derived neurons or in neuronal cell lines with the requisite sensitivity. Most of these assays require a secondary detection method where cell extracts are prepared after toxin treatment and probed for BoNT cleavage products using protein blotting or ELISA-based methods [32,33,35,37]. Thus, in addition to selecting and optimizing culture conditions to maximize assay sensitivity and robustness, these methods require the development and characterization of antibodies that are specific and sensitive to BoNT substrate cleavage products. Alternatively, secondary detection methods can be avoided by transfecting cells with reporters that detect BoNT activity. For example, Dong et al. describe the use of fluorescent reporters to detect intra-cellular BoNT activity [30]. These reporters contain sequences of SNAP-25 or VAMP fused to cyan (CFP) and yellow (YFP) fluorescent proteins. Cleavage of the reporters by BoNT results in a change in Förster resonance energy transfer (FRET) emissions between CFP and YFP. While an elegant solution for monitoring BoNT activity without secondary cell processing and detection, the assay requires the transient transfection of cells with the reporters, as well as low-throughput fluorescent microscopy techniques to detect and quantify the FRET signal.

In these studies, a CBPA, called BoSapient™, for determining the relative potency of BoNT/A-containing drug products and substances is described. The BoSapient cell line stably expresses a fluorescent reporter that enables the detection of BoNT/A using a fluorescent plate reader without any post-treatment processing other than washing the assay plates before collecting fluorescence emissions. The studies demonstrate that the BoSapient CBPA has the requisite properties to account for all of BoNT’s cellular activities, has sensitivity appropriate for BoNT/A-based drug products, is precise and accurate over a range BoNT concentrations, and is suitable for long-term use. Finally, the assay can be adapted to semi-automation, yielding a method with excellent precision and accuracy while reducing operator dependency.

## 2. Results

### 2.1. Description and Operation of the BoSapient CBPA

**Figure 1:**
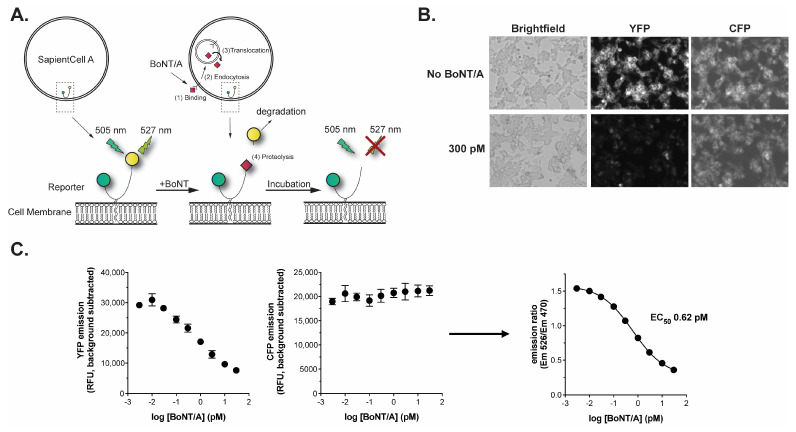
The BoSapient A CBPA. (**A**) Overview of the BoSapient CBPA. The CBPA uses an engineered human cell line that stably expresses a protein reporter containing CFP (green circle) and YFP (yellow circle) connected by full-length SNAP-25 and targeted to the cellular membrane. Refer to the main text for additional details. (**B**) BoSapient cells show reduced YFP emissions but not CFP emissions following incubation with BoNT/A. Cells were incubated in SAM with or without 300 pM BoNT/A for 48 h before imaging using an InCell Analyzer microscope. (**C**) Fluorescent emissions of the BoSapient CBPA in response to BoNT/A. The CBPA was executed as described in Section 4 with the indicated BoNT/A concentration and using a fluorescent plate reader to capture YFP and CFP responses. Background-subtracted YFP (**left**) emissions are divided by the background-subtracted CFP (**center**) emissions for a given assay plate well to generate an emission ratio (**right**). The emission ratio values were plotted as a function of BoNT/A concentration and fitted using a 4-parameter logistic (4PL) regression. Error bars represent the standard deviation of the mean. Limits of detection and quantitation were calculated from the mean emission ratio of the negative control wells minus 3 × standard deviation (SD) or 6 × SD, respectively.

The BoSapient cell line is an engineered human neuroblastoma cell line that stably expresses a reporter consisting of full-length SNAP-25 expressed between N-terminal CFP and C-terminal YFP moieties (Figure 1A). Upon expression, the reporter is targeted to and associates with the plasma membrane through palmitoylation of cysteine residues in SNAP-25. In the absence of toxins, excitation of the cells at ~505 nm results in strong YFP emission at ~527 nm. When BoNT/A is present, the toxin (1) binds to cell-surface receptors and (2) is internalized through endocytosis. The BoNT/A light chain (Lc/A) then (3) translocates into the cytosol, where it then (4) cleaves the reporter’s SNAP-25 sequence, releasing a fragment containing residues 198–206 of SNAP-25 and the YFP moiety. This fragment is then rapidly degraded by the cell, resulting in a decrease in YFP emissions that is visualized by fluorescence microscopy (Figure 1B) or measured on a fluorescent plate reader. The CFP-containing fragment remains bound to the plasma membrane and is not degraded, allowing for the normalization of the decrease in the YFP signal based on protein expression at the site of measurement. The reporter readout is notably different than that shown by Dong et al., where decreases in FRET between YFP and CFP were used to detect BoNT activity [30]. Here, BoNT activity is detected solely by the loss of directly excited YFP emissions.

The BoSapient CBPA is operated in a 96-well plate format where cells are seeded into an assay plate and incubated overnight before the application of samples. After ~48 h incubation with the samples, the plate is washed with PBS to reduce background and CFP and YFP emissions are collected using a fluorescent plate reader (Figure 1C). The decrease in YFP fluorescence is BoNT/A dose-dependent and is directly proportional to the neurotoxin’s biological activity, while the CFP emissions exhibit low BoNT dependency. Calculating the emission ratio (background-subtracted YFP/background-subtracted CFP) normalizes the reduction in YFP emission at the site of the measurement (Figure 1C). Upon application of a suitable dose-range of BoNT/A, a sigmoidal dose–response is achieved with a BoNT/A EC_50_ of <1 pM (<150 pg/mL for BoNT/A) and limits of detection and quantitation of 0.03 pM (4.5 pg/mL) and 0.11 pM (16.5 pg/mL), respectively. This sensitivity is suitable for the detection and quantification of BoNT/A-based drug products.

### 2.2. BoNT/A Receptor Characterization and Receptor Dependency of the BoSapient Cell Line and CBPA

**Figure 2:**
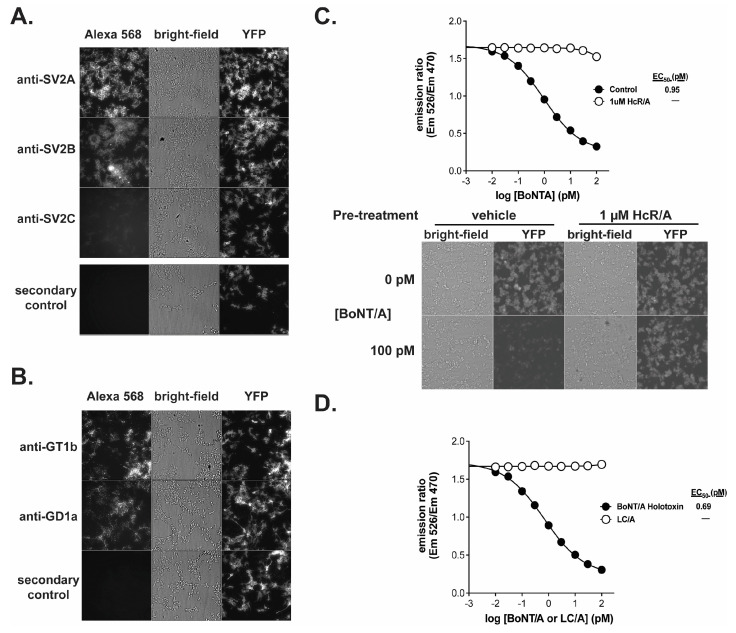
The receptor binding domain of BoNT/A is required to elicit a response in the BoSapient CBPA. Cells were fixed and stained against SV2 isoforms SV2A, SV2B, and SV2C (**A**) or GD1a and GT1b (**B**) and imaged as described in Section 4. (**C**) A CBPA was executed as described in Section 4 except that seeded assay plates were pre-treated without (control) or with 1 µM recombinant BoNT/A heavy-chain (HcR/A) in assay media for 1 h at 37 °C before treatment with serial dilutions of BoNT/A holotoxin for 48 h at 37 °C. Emission ratios are plotted as a function of BoNT/A concentration (**top**). Bright-field and fluorescent images are shown for representative wells treated with 0 or 100 pM BoNT/A (**bottom**). (**D**) The CBPA was executed as described in Section 4 except assay plates were treated with serial dilutions of either BoNT/A holotoxin or BoNT/A light-chain (LC/A) for 48 h at 37 °C. Emission ratios are plotted as a function of BoNT/A or LC/A concentration.

The cell line was characterized by immunofluorescence for the presence of receptors required for BoNT/A uptake (Figure 2). Synaptic Vesicle Protein 2 (SV2) and complex gangliosides are cellular co-receptors for BoNT/A which bind two distinct regions of the HcR/A domain [10,42,43]. Cells express SV2 isoforms A and B but do not strongly express SV2C as judged by immunofluorescence (Figure 2A). However, expression of any one of the three SV2 isoforms is sufficient for BoNT/A uptake [10]. The cell line also strongly expresses the complex gangliosides GD1a and GT1b (Figure 2B), both of which were previously shown to bind BoNT/A and support subsequent BoNT/A intoxication [44,45,46,47]. Therefore, the cell line expresses the co-receptors necessary for BoNT/A uptake.

The receptor dependency of BoNT/A uptake by the BoSapient CBPA was evaluated in two functional tests. First, BoNT/A was applied to the CBPA in the presence and absence of recombinant HcR/A, which can bind cellular receptors but lacks proteolytic activity [48]. If receptor binding is required for BoNT/A-dependent responses in the CBPA, the addition of HcR/A will compete with BoNT/A and decrease the toxin’s activity in the CBPA. As shown in Figure 2C, addition of 1 µM HcR/A inhibited BoNT/A activity in the CBPA across all concentrations tested, including BoNT/A concentrations that saturate the assay in the absence of HcR/A. There is no decline in emission ratio values when 1 µM HcR/A is combined with increasing concentrations of BoNT/A. In addition, microscopic images show that YFP emissions are maintained when the cells are incubated with 100 pM BoNT/A and 1 µM HcR/A but not when 1 µM HcR/A is replaced with vehicle. For the second functional test, recombinant LC/A, a BoNT/A fragment that exhibits proteolytic activity but lacks the ability to bind cellular receptors, was applied to the CBPA (Figure 2D). While a dose–response curve was fully developed with BoNT/A, LC/A did not elicit a response in the assay at any of the tested concentrations. These results indicate that the BoSapient CBPA only measures the activity of fully competent BoNT/A, making it a suitable replacement for the MBPA.

### 2.3. Cell Line Storage and Passage Stability

Long-term (>10 years) use and efficacy of a CBPA is managed by generating master and working cell banks of the cell line stored in liquid nitrogen [49]. The master cell bank serves as a permanent source of the cells and for producing working cell banks that then supply cells for day-to-day use. Vials of a given bank are thawed and passaged to propagate the cells for use. A key attribute of any CBPA cell line is the ability of the banked cells to be robustly thawed and passaged 20 or more times without loss of cell health, yield, or CBPA performance.

**Figure 3:**
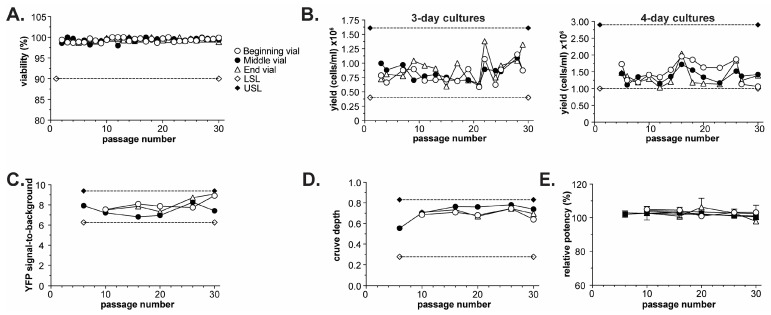
Passage stability of the BoSapient cell line. Three vials, filled at the beginning (3/300), middle (151/300), or end (297/300) of a 300-vial master cell bank, were thawed from liquid nitrogen and passaged every 3–4 days (see Section 4). Every 5 ± 2 passages, 4-day cultures were subjected to a CBPA, as described in Section 4. (**A**) Cell viability during passaging at the indicated passage number for cultures derived from the three vials. A lower specification limit (LSL) of 90% was applied to the study. (**B**) Cell yields for 3- (**left**) and 4-day (**right**) cultures at the indicated passage number for cultures derived from the three vials. LSL and upper specific limit (USL) are based on yields obtained from the cell line during development. (**C**) YFP signal-to-background from execution of CBPAs as a function of passage number for all three vials. YFP signal-to-background was calculated by dividing the YFP emissions from assay plate wells containing cells but no BoNT/A (negative control) by the YFP emissions from wells containing no cells (blank). LSL and USL are calculated from mean from all CBPA runs ± 20%. (**D**) CBPA curve depth as a function of passage number for all three vials. Curve depth was calculated by subtracting the emission ratio (assay response) at the highest BoNT/A concentration from the emission ratio at the lowest BoNT/A concentration. LSL and USL are set as the beginning curve depth (passage 6) ± 50%. (**E**) CBPA relative potency results as a function of passage number and vial.

A 300-vial master cell bank of the BoSapient cell line was generated and then subjected to passage stability testing. Three vials, filled at the beginning (3/300), middle (151/300), or end (297/300) of the banking process, were thawed and passaged 30 times while monitoring cell viability and yield over the course of the ~4-month study (Figure 3). Cell viability was >95% across all 30 passages (Figure 3A). The cell line was routinely passaged every 3 or 4 days, and yields—while variable—fell within historical limits obtained during cell line development (Figure 3B). Furthermore, when each vial’s growth curve was fit by linear regression, the resulting slopes were not found to be significantly non-zero (*p* < 0.05, required for significance). Thus, the cell line was found to be growth-stable for at least 30 passages.

The performance of the cell line was tested by executing a CBPA every 4–6 passages during the 30-passage study using cells from each of the three banking positions. For each assay, serial dilutions of a reference and test sample generated from the same BoNT/A holotoxin lot were applied to the CBPA. Test sample potencies were then determined relative to the reference sample using parallel line analysis (see Section 4). Performance attributes, including fluorescence signal-to-background (reporter stability), assay curve depth (dynamic range and sensitivity stability), and test sample relative potency (functional stability), were collected and plotted as a function of passage number (Figure 3C–E).

The YFP signal-to-background was largely unchanged from the beginning to the end of the study, with all values falling within 20% of the mean (Figure 3C), indicating that the cell line does not substantially lose reporter expression over the course of 30 passages. Similarly, assay curve depth (assay response_lowest [BoNT/A]_ – assay response_highest [BoNT/A]_) was within 50% of the beginning curve depth (passage 6) and did not significantly change over the course of 30 passages (Figure 3D). Curve depth consistency demonstrates that the BoSapient CBPA’s dynamic response and sensitivity to BoNT is similar over 30 passages. Finally, all 32 test sample relative potency results were within 97.1–106.2% (100% expected), with all associated SD values < 4 for a given passage number, indicating excellent accuracy and precision for a CBPA (Table 1). The relative potency data were also plotted as a function of passage number and vial banking position (Figure 3E). No significant passage-dependent or vial-dependent effects were noted for the study; upon completing a linear regression of the data, all slopes were found not to be significantly non-zero (*p* = 0.352, 0.281, and 0.341 for beginning, middle, and late vials, respectively). This data suggests that the cell line’s health, cell yields, and CBPA performance is not substantially affected by passaging up to 30 times, making the BoSapient cell line suitable for use in a CBPA. The generated master cell bank exhibited uniform performance across vial fill positions with extended passaging and can thus be used as a source of consistent cells for many years.

### 2.4. Manual and Semi-Automated Methods for Relative Potency Testing of BoNT/A Samples

Relative potency methods using cell-based assays are often executed as parallel-line assays (PLAs) where the doses/concentrations applied to the CBPA are chosen to elicit responses within the assay’s linear range [50]. BoNT concentrations in the asymptotes of the dose–response curve, where doses yield either limited responses or saturating responses, are avoided. This contrasts with the alternate means of performing a relative potency method where samples are diluted over a large concentration range such that the three phases of the given assay—limited response, linear response, and saturated response—can be fit with a non-linear regression such as 4-parameter logistic (4PL). Saturating a CBPA with a BoNT/A-based drug product can be challenging because of the low BoNT/A concentrations contained within a given drug product vial (1–12 ng per vial) and due to the presence of excipients used to stabilize biotherapeutics. These excipients, which may include sugars, salts, proteins, peptides, or surfactants, can affect a CBPA’s end-of-assay cell health and/or sensitivity. For these reasons, a PLA-based approach, where saturating drug product doses are not required, is often the preferred method when designing a CBPA for BoNT/A-based drug products. Furthermore, this approach is advocated by regulatory guidelines when appropriate [49].

**Figure 4:**
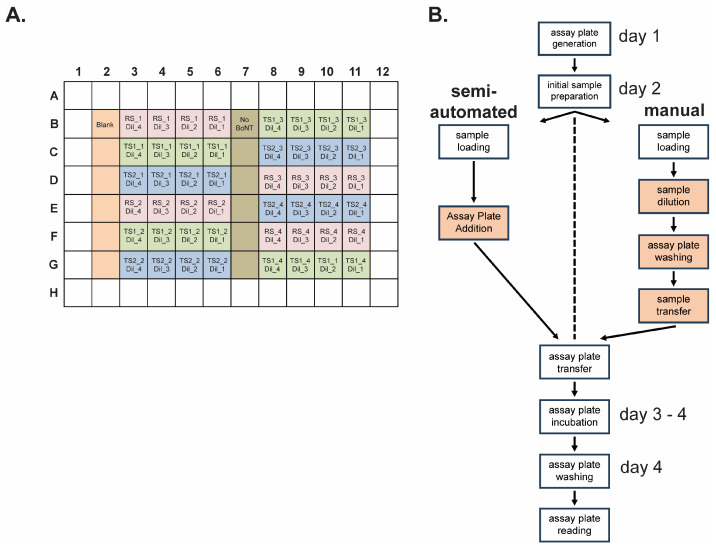
Semi-automated and manual operation of relative potency methods using the BoSapient CBPA. (**A**) Final assay plate layout for both the manual and semi-automated methods. Reference sample (RS, pink), test sample 1 (TS1, green), and test sample 2 (TS2, blue) serial dilutions (i.e., Dil_1, Dil_2, Dil_3, Dil_4) are applied in quadruplicate (e.g., RS-1, RS-2, RS-3, and RS-4) with each replicate independently diluted in parallel. Wells containing no cells (Blank, orange) or cells with no BoNT/A (dark green) are included as controls. Replicates are arrayed to minimize plate effects. (**B**) Workflow of the semi-automated and manual CBPA methods. Operator-performed actions for each method are shown in boxes. Both methods share cell maintenance, assay plate seeding, and initial sample preparation/suspension procedures but diverge during sample serial dilution, assay plate washing, and sample application procedures; these actions are fully automated in the semi-automated method. Thus, the operator only needs to insert the assay plate into the platform when prompted. Operator-performed steps that diverge between the manual and semi-manual methods are highlighted in orange.

The PLA methodology used for the BoSapient CBPA requires that each sample, both reference and test, be serially diluted to four concentrations that are then applied to the CBPA plate (Figure 4A). The concentrations chosen should consistently yield responses in the BoSapient CBPA’s linear range. For the BoNT/A internal standard used here, the chosen doses are 0.5, 1, 2, and 4 pM, corresponding to 0.075, 0.15, 0.3, and 0.6 ng/mL BoNT/A holotoxin (MW = ~150 kDa), respectively. Each sample is serially diluted in quadruplicate and in parallel on a staging plate and then arrayed onto a pre-seeded assay plate in a pseudo-randomized fashion to minimize plate effects. The final assay plate contains quadruplicate dilutions from one reference and two different test samples (Figure 4A), yielding two relative potency determinations per plate.

Manual and semi-automated BoSapient CBPA methods were developed for execution of a relative potency method for BoNT/A-containing samples (Figure 4B). Both methods contain the same procedural outcomes and time course: On day 1, cells from a 4-day culture are plated on an assay plate and grown overnight. On day 2, serial dilutions of the test and reference samples are generated, the assay plate is washed to exchange the growth media, and the diluted samples are transferred to the assay plate. The assay plate is then incubated for two days before washing the plate and collecting the fluorescence emissions on day 4. Both the manual and semi-automated methods require the operator to formulate media and reagents, to complete any initial sample preparation (e.g., vial suspension), and to perform cell passaging and assay plate seeding procedures. Both methods also share procedures for final assay plate washing with a programable 96-well plate washer and plate reading using a fluorescence plate reader. These shared tasks require standard laboratory and cell passaging skills and, while important, have less impact on overall assay precision.

The manual and semi-automated methods diverge on day 2, specifically during sample serial dilution, assay plate washing, and assay plate sample application, steps which are more critical for assay precision and require additional operator training for optimal results. In the manual method, these steps are performed by hand using computerized multichannel pipets. The semi-automated method uses an automated pipetting platform that allows for additional precision and control over pipetting parameters. These parameters include aspiration and dispensing speed, sample volume, and X-, Y-, and Z-positioning for within-well placement of the pipette tips. Furthermore, the platform has 12 positions for buffer reservoirs, pipette tips, plates, and any required adapters to complete the tasks. The automated pipetting platform is operated by a software script developed by the end-user to complete the necessary tasks.

A script for executing the BoSapient CBPA was developed and all parameters were optimized to minimize cell loss during aspiration and dispensing steps and to precisely perform pipetting tasks with minimal sample loss and carry-over between wells. The finalized script was then validated to demonstrate that the scripted commands result in the platform executing all intended and coded tasks, and that volumes from aspiration and dispensing steps meet expectations for accuracy and precision. An account of the script validation can be found in the Supplementary Materials S2. The semi-automated method was then qualified and compared to the manual method.

### 2.5. Manual and Semi-Automated Performance of the BoSapient CBPA

The semi-automated and manual methods were qualified in parallel to demonstrate the performance of each method and to determine whether incorporating automation into the CBPA method yielded any performance gains. Each method was qualified according to pharmaceutical regulatory guidelines for accuracy, precision, linearity, and range [51,52,53]. The qualification was completed by formulating test samples to 64, 80, 100, 125, and 150% of a 4 pM nominal concentration (i.e., 80–120% of BoNT/A-based drug product release specifications) and assaying each sample concentration six times with each method. Intermediate precision was evaluated by varying testing days and by using two different operators for the manual method or two different pipetting heads for the semi-automated method. Calibration of the automated pipetting platform is entirely contained within the interchangeable pipetting head, so testing two heads demonstrates the performance expected between two independent automated pipetting platforms. Acceptance criteria were based on the typical release specifications of a BoNT-based drug product where the potency should be within 80–125% of the labeled potency [54]. A test schedule and qualification acceptance criteria can be found in Supplementary Materials S1.

**Figure 5:**
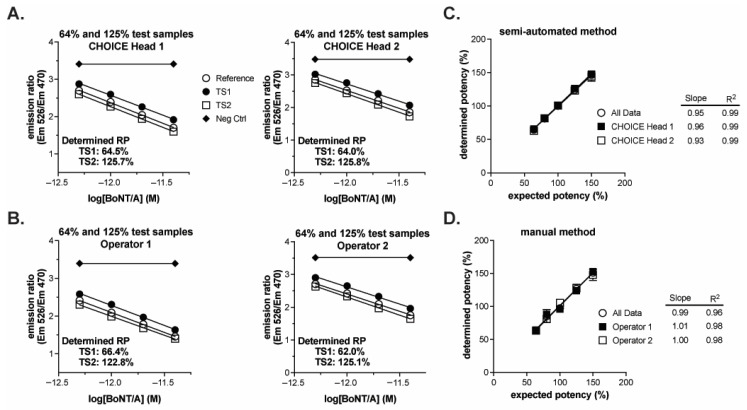
Qualification of semi-automated and manual BoSapient CBPA methods. CBPAs were executed as described in the Experimental Procedures section. Test samples were formulated to 64, 80, 100, 125, or 150% of a 4 pM nominal concentration, corresponding to 80–120% of a typical 80–125% drug product release specification. (**A**) Example CBPA runs from the semi-automated method qualification. Assay plates were run independently with different pipetting heads fitting the automated pipetting platform. The theoretical potency of the two test samples were 64 and 125% and each was run against a 100% reference. Emission ratio was plotted as a function of BoNT/A concentration, assuming 100% nominal concentrations. (**B**) Example CBPA runs from the manual method qualification. Assay plates contain test samples of the same theoretical potency as described in (**A**), but the assays were executed independently and manually by two different operators. (**C**) Relative potency results from the semi-automated method qualification plotted as a function of expected/theoretical concentration. Each data point represents the mean and SD of six independent determinations. Data were fit by linear regression. (**D**) Relative potency results from the manual method qualification plotted as a function of expected/theoretical concentration. Data were treated as described in (**C**).

A summary of the results is provided in Table 2, and the complete set of individual results is found in Table S1. Execution of both methods yielded similar data regardless of operator or pipetting head. When over-potent (e.g., 125% of nominal concentration) or under-potent (e.g., 64% of nominal concentration) test samples were applied to the CBPA along with a 100% reference sample, the dose–responses of the test samples were right (under-potent) or left (over-potent) shifted relative to the reference sample dose–response for both the semi-automated (Figure 5A) and manual (Figure 5B) methods. Upon replicate testing of all sample concentrations, the determined potencies were proportional to the expected potencies for both methods (Figure 5C,D). Both methods yielded linear data with slopes at or near one (perfect agreement between determined and expected) with the manual method yielding data with a slope slightly closer to the ideal, indicating slightly better accuracy over the range of sample concentrations (slopes = 0.95 and 0.99 for semi-automated and manual methods, respectively). The data from the semi-automated method, however, yielded a higher R^2^ value, indicating improved precision across the test sample concentration range tested (R^2^ = 0.99 and 0.96 for semi-automated and manual methods, respectively). Furthermore, the root mean square error for the linear regression was 4.47 for the manual method and 2.95 for the semi-automated method. The improved precision of the semi-automated method is also evidenced by the SD of the mean determined potencies for each test sample concentration: The semi-automated method yielded SDs of 1.9–4.9, with a mean of 2.6, while the manual method yielded SDs of 1.6–5.3, with a mean of 4.3 (Table 2). Both methods yield acceptably precise results as further described below; however, the semi-automated method yields more precise results.

**Figure 6:**
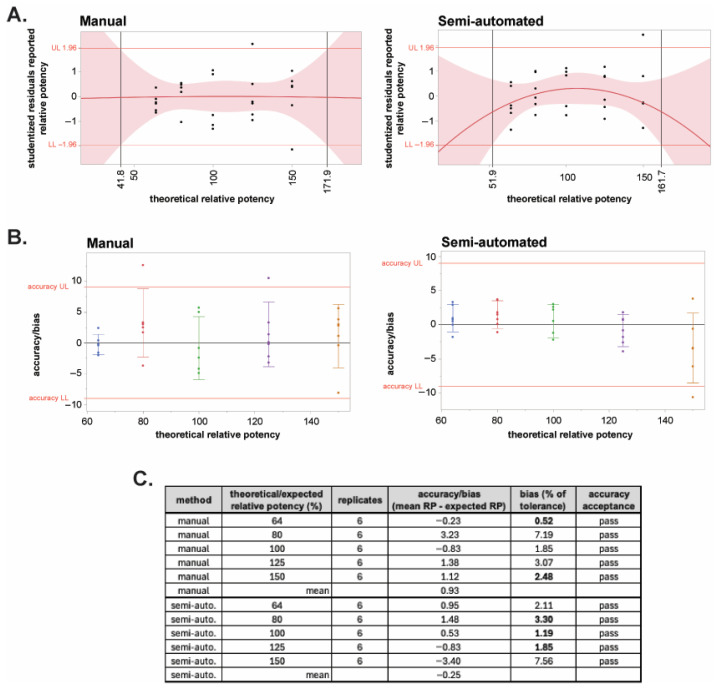
Linearity and bias of the semi-automated and manual BoSapient CBPA methods. (**A**) Quadratic plots of the studentized residuals of all results of the manual (**left**) and semi-automated (**right**) method qualifications were constructed as a function of theoretical relative potency and used to establish the linear range of the methods. Red shading represents the limits of the 95% confidence interval (CI). A studentized residual limit of ±1.96 was used to determine the upper (UL) and lower (LL) limits of the assay’s linear range. (**B**) Accuracy or bias, the difference between the mean measured RP value and the expected RP value at each tested theoretical concentration, was determined for the manual (left) and semi-automated (right) method qualification data and plotted versus theoretical potency. Based on typical 80–125% drug product specification limits, a bias limit of ±9 was calculated (20% of tolerance). (**C**) Accuracy results and acceptance status for all tested concentrations in the two methods.

The linearity of the two methods was evaluated by constructing a quadratic plot of the studentized residuals as a function of the expected/theoretical potency for each method (Figure 6A). Limits were set at ±1.96 (95% certainty) of the studentized residuals. The quadratic fit is used to evaluate the lack of linearity or the presence of curvature in the assay: The points where the 95% CI of the curve exceeds ±1.96 are where the methods exhibited excessive curvature. The concentrations within those points determined the linearity of the method. Based on these criteria, the linearity is from 41.8 to 171.9% for the manual method and 51.9 to 161.7% for the automated system (Figure 6A). These results indicate that both methods can quantify samples outside of the range of sample concentrations tested and that the manual method may be able to quantify a larger range of sample concentrations. Linearity should, however, be restricted to the range of concentrations tested; therefore, the linearity for both the manual and automated systems is 64% to 150% (2.56 to 6 pM) of the nominal test sample concentration of 4 pM, and both methods satisfy the acceptance criteria for linearity.

Accuracy/bias expresses the closeness of agreement between the mean measured value and the expected value at each theoretical dilution tested. The acceptance criterion for accuracy is the bias as a percentage of the product specification tolerance (80–120%). Typically, for bioassays, if the bias as a percentage of tolerance is ≤20%, then the assay is deemed accurate at a given test sample concentration [51]. Figure 6B shows the bias for each theoretical concentration for both the manual and semi-automated methods. At all five theoretical concentrations, both methods pass the acceptance criterion of ≤20% of tolerance (Figure 6C). The bias of the automated method was lower across all concentrations except 64 and 150% as visualized by less data scatter (Figure 6B) and as quantitated as bias as a percentage of the tolerance for each concentration (Figure 6C). The determined mean accuracy for manual and semi-automated methods was 0.93 and −0.25, respectively. While both methods pass the criteria for accuracy, the semi-automated method exhibited improved accuracy compared to the manual method.

Repeatability and intermediate precision were determined using partition of variance (POV) analysis to isolate the variance components for each method (manual or semi-automated). The variance component of operator/head is the between-main-effect variation (inter-assay error) in the model. Repeatability is based upon the within-assay variation (intra-assay error). Intermediate precision is calculated using the standard deviation of all assay results at each theoretical dilution. The analysis procedures and acceptance criteria for repeatability and intermediate precision were previously described [51,52]. To pass the acceptance criteria for repeatability and intermediate precision, the results for all theoretical dilutions must be below the percent of tolerance limit. The repeatability acceptance criterion is ≤60% of tolerance; the intermediate acceptance criterion is ≤70% of tolerance.

When using an *n* = 1 independent determinations assumption for each theoretical dilution, both the manual and semi-automated methods passed the repeatability acceptance criterion of ≤60% of tolerance (Table 3). The mean repeatability for the manual method is 2.75% and that for the semi-automated method is 2.12%, indicating slightly improved precision with automation. When using an *n* = 1 independent determinations assumption for each theoretical dilution, both the manual and semi-automated methods passed the intermediate precision acceptance criterion of ≤70% of tolerance (Table 3). The semi-automated method had lower intermediate precision across all concentrations except the 64% sample. The mean intermediate precision is 3.95% and 2.42% for the manual and semi-automated methods, respectively. A ~39% reduction in method error is demonstrated by the semi-automated method, indicating that the semi-automated method is more stable during day-to-day operation.

The final method qualification parameter is range. A method’s range is established when the assay is determined to be accurate, repeatable, precise, and linear. The assay range is set based on the maximum concentration range that meets all acceptance criteria for the validation attributes. Since all acceptance criteria were met at all tested concentrations, the maximum range is 64 to 150% of the nominal concentration for both methods.

**Figure 7:**
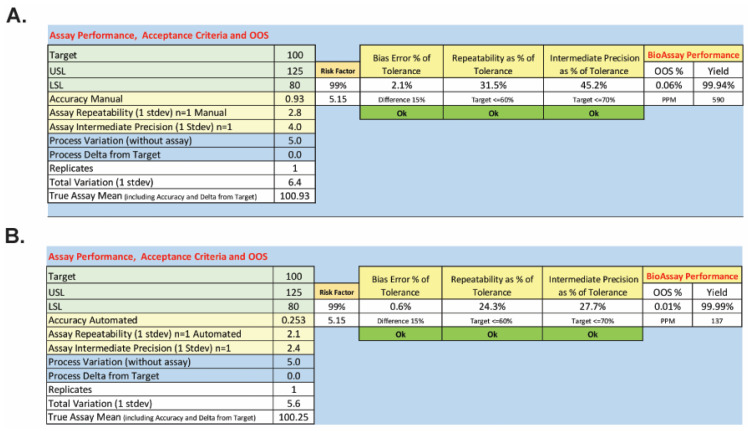
Predicted OOS rates for the manual (**A**) and semi-automated (**B**) BoSapient CBPA methods. To determine the out-of-specification (OOS) rate as a percentage or in parts per million (PPM), the mean including analytical bias and the associated method standard deviation was determined from the method qualification data. Applying lower (80%, LSL) and upper (125%, USL) specification limits, the area under the normal curve outside of the specification limits was then calculated using the following equations: %OOS = (1 − NORMSDIST((Mean − USL)/standard deviation) + NORMSDIST((Mean − LSL)/standard deviation)) × 100 and OOS rate PPM = (1 − NORMSDIST((Mean − USL)/standard deviation) + NORMSDIST((Mean − LSL)/standard deviation)) × 1 × 10^6^.

Both the manual and semi-automated methods pass qualification, but the semi-automated method yielded data with improved accuracy and precision relative to the manual method. These improvements were determined to be significant by Bartlett’s F-test upon evaluation of the bias of each method and its repeatability (see Supplementary Materials S3). To consider the practical implications of the improvements in performance, the mean, bias, and precision for each method were used to calculate the expected out-of-specification (OOS) rate based on all measured error sources. Figure 7 shows the parts per million (PPM) that could be OOS and predicts the overall OOS rate based on the qualification results and normal operations of the BoSapient CBPA using the manual and semi-automated methods. The manual method yielded, due to error, a predicted PPM of 590 and an OOS rate of 0.06%; the semi-automated method yielded a predicted PPM of 137 and an OOS rate of 0.01%. While both methods would be expected to perform well, the predicted OOS rate for the semi-automated method is ~sixfold lower than that of the manual method.

### 2.6. Testing of Commercially Available Drug Products

The above studies were completed, due to cost and availability, with an internal standard formulated from research-grade, purified BoNT/A holotoxin (150 kDa), and excipients commonly found in commercial drug products (see Section 4). To further demonstrate its utility, the semi-automated method was executed using samples composed of BOTOX^®^ (OnabotulinumtoxinA, AbbVie, North Chicago, IL, USA) and Xeomin^®^ (IncobotulinumtoxinA, Frankfurt, Germany), commercial drug products containing the 900 kDa complexed and 150 kDa purified BoNT/A forms, respectively [55]. Each drug product was suspended and then diluted to 100% nominal concentrations of 30 or 40 U/mL, respectively. The 100% nominal reference samples were then used to determine the potency of test samples diluted to theoretical concentrations of 80 and 125% of the nominal concentration. The samples were subjected to the semi-automated method, and the relative potencies were determined.

**Figure 8:**
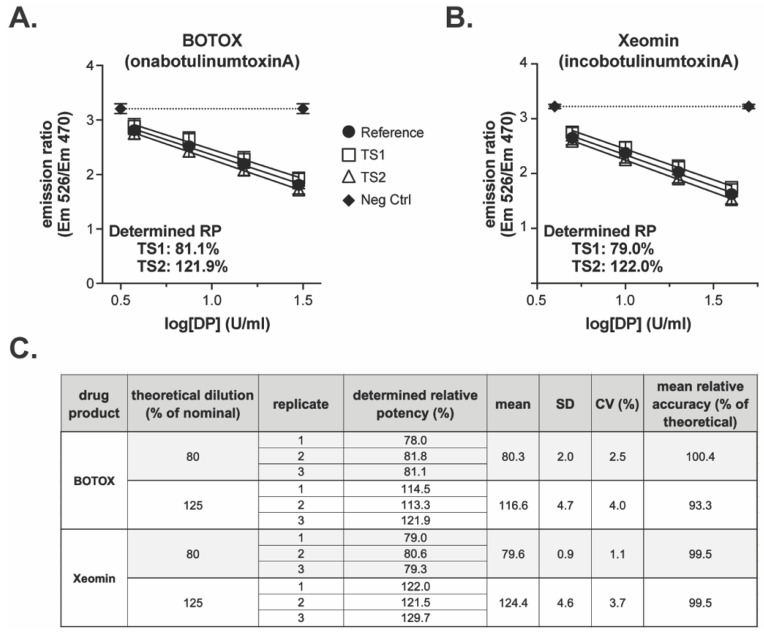
Testing of commercial drug product with the semi-automated BoSapient CBPA method: CBPAs were executed as described in Section 4. Test samples were formulated to 80 or 125% of a 30 U/mL (BOTOX) or 40 U/mL (Xeomin) nominal concentration. Reference samples were formulated to 100% of the nominal concentration. Example CBPA runs for BOTOX (**A**) and Xeomin (**B**) are shown. Emission ratio was plotted as a function of drug product concentration assuming 100% nominal concentrations. (**C**) Relative potency results from three replicate CBPA runs of the semi-automated method for both BOTOX and Xeomin.

Both drug products elicited responses in the BoSapient CBPA method and exhibited differentiation between the 80 and 125% test samples when compared to the 100% reference samples (Figure 8). The determined relative potencies for the representative BOTOX event were 81.1 and 121.9% for the 80 and 125% samples, respectively (Figure 8A). For Xeomin, the relative potencies determined were 79.0 and 122.0%, respectively (Figure 8B). A total of three replicates were completed for each drug product, yielding overall means of 80.3 and 116.6% for the 80 and 125% samples of BOTOX and 79.6 and 124.4% for the Xeomin samples (Figure 8C), and these values were all within 10% relative accuracy of the theoretical concentrations. Method precision was 4% CV or less across all test samples. The data in this supporting study indicate that the semi-automated CBPA method is suitable for the relative potency testing of commercial drug products.

## 3. Discussion

A number of CBPAs for BoNT/A are described that rely, generally, on one of three methods for detecting BoNT/A activity: (1) detection of cellular secretion products or post-synaptic processes [38,39,40]; (2) detection of native SNAP-25 cleavage using a secondary assay such as ELISA [32,33,34,35,37]; or (3) detection of response changes (e.g., fluorescence) of recombinant reporters that are cleaved by BoNT/A [30,31]. When considering a CBPA for the testing and release of BoNT-based drug products, the mode of detection affects its utility and performance. Detection mode 1 is currently limited to research settings as the published CBPAs rely on primary neurons and/or the use of low-throughput microscopic or electrophysiological detection methods. Detection mode 2 requires the additional development of the secondary detection method which may include antibody development. Furthermore, the throughput of these CBPAs is limited by the cell processing required after incubation of the cells with BoNT/A-containing samples, followed by the execution of an immunoassay to detect the cleavage of SNAP-25. As to sources of error for detection mode 2, these CBPAs will be sensitive to errors associated with both the dilution and application of samples, as well as the error associated with cell processing and ELISA methodology.

CBPAs that use detection mode 3, such as the BoSapient CBPA described here, have a significant advantage over other CBPAs in that no secondary detection is required. The output of the BoSapient CBPA is obtained by a straightforward collection of fluorescence emissions. This increases the throughput of the CBPA and eliminates compounding sources of error. Furthermore, the BoSapient CBPA measures BoNT/A activity by direct excitation of YFP and is not reliant on low-efficiency techniques such as FRET or downstream activation of a secondary reporter gene. To successfully adapt this detection mode, significant time (1–2+ years) and resources are necessary to engineer and develop an appropriate cell line that stably expresses the reporter. However, once the cell line is characterized, banked, and optimized for use within a CBPA, a sensitive and robust methodology can be established.

Developing a CBPA that is appropriate for long-term testing and release of BoNT/A-based drug products requires more than just a robust detection methodology. The CBPA must be shown to detect only fully functional BoNT/A and account for the toxin’s mechanism of action [56,57]. The studies described here demonstrate that the BoSapient cell line expresses the BoNT/A co-receptors SV2A, SV2B, GT1b, and GD1a; that the BoSapient CBPA only responds to the LC/A that is part of the entire BoNT/A protein; and that BoNT/A activity is disrupted by competitive inhibition of receptor binding activity. The BoSapient CBPA only responds to intact and functional BoNT/A.

A CBPA suitable for drug product testing requires the establishment of vial banks from which cells are thawed and passaged without loss of cell line integrity or CBPA performance. Without this, robustness issues may arise where CBPA performance cannot be sustained over time, potentially disrupting product release. Demonstration of such stability is largely devoid from publications describing other BoNT-sensitive CBPAs. Here, the BoSapient cell line was successfully banked, and the resulting frozen cells were thawed, grown, and used in a CBPA over at least 30 passages (3–4 months) per vial without loss of performance. Thus, a single bank of 300 vials could supply the BoSapient CBPA for decades with periodical re-testing.

Finally, a CBPA must be able to determine the relative potency of test samples with sufficient accuracy and precision to meet regulatory guidelines [49,52,53]. Only two other CBPAs are publicly described as having sufficient performance for regulatory acceptance as a commercial release assay for a BoNT-based drug product [31,32]. Other CBPAs described for BoNT lack detailed demonstrations of repeatability across a range of test sample concentrations. Here, the described BoSapient CBPA was incorporated into manual and semi-automated methods for relative potency testing of BoNT/A-containing samples, and both methods were qualified according to regulatory guidelines. Both methods were found to be linear, accurate, and precise over a range of test sample concentrations. Furthermore, the method was also found to be accurate and precise with two different commercial drug products.

Comparability testing between the CBPA and MBPA will be required for regulatory acceptance of the BoSapient CBPA. Such studies are not included here due to the time, cost, and animal lives required to develop an MBPA method specific to the BoNT/A samples described here. A comparability study would then need to be completed between the developed MBPA and BoSapient CBPA methods. It should be noted that MBPA methods are not universal and are specific to both sample/drug product composition and potency as well as the laboratory executing the method. Likewise, comparability studies are also method- and drug product-specific because each drug product will differ in formulation, potency, and/or stability. The studies described here, however, demonstrate that BoSapient CBPA meets regulatory requirements for precision and accuracy.

The final outcome of this study is the first demonstration of a semi-automated method for the relative potency testing of BoNT/A-containing samples. Incorporating automation into the CBPA for a regulated environment significantly reduces the training required and the potential for elevated inter-operator and inter-laboratory variability [58]. The steps that are automated—sample serial dilution, assay plate washing, and sample application—are tasks that require operator expertise and continual training to minimize cell loss, maintain assay performance, and provide day-to-day and laboratory-to-laboratory consistency. While these tasks are, generally, easily completed by experienced and qualified analysts, inter-operator variation will always exist due to operator-to-operator differences in pipetting, dilution, and cell handling techniques and normal work fatigue. These sources of error can be compounded if the operators are not well trained or overworked. Automation of precision-critical steps reduces these sources of error while minimizing training requirements. Full automation of the CBPA would drastically increase automation complexity and cost due to the need to integrate robotics with other equipment including incubators, plate washers, and plate readers. Therefore, these steps were not automated.

Semi-automation of the BoSapient CBPA also reduces the potential for disruption of drug product commercial release through reduction in OOS rates and ease of transfer. A ~39% error reduction in intermediate precision was observed for the semi-automated method compared to the manual method, resulting in a sixfold reduction in predicted OOS rates for the semi-automated method. Here, the manual method was executed by two operators with more than three years’ experience with the CBPA. The potential error reduction would be even greater in environments where the operators have less experience. In contrast, intermediate precision of the semi-automated method was determined using two different pipetting heads on the automated pipetting platform. Since all critical calibration is contained within the heads, the low intermediate error indicates that a laboratory can adopt the method to multiple machines either in the same or different facilities and expect similar performance. Thus, semi-automation of the BoSapient CBPA will improve comparability between testing sites, increasing overall method robustness and reducing potential commercial disruption.

## 4. Materials and Methods

### 4.1. Materials and Reagents

The BoSapient cell line was provided by BioSentinel, Inc. (Madison, WI, USA). RPMI 1640, GlutaMAX™, non-essential amino acids, HEPES, sodium pyruvate, trypsin-EDTA, trypan blue, and B-27™ supplement were provided by Thermo Fisher (Waltham, MA, USA). Fetal bovine serum was obtained from ATCC (Manassas, VA, USA). iBAM2 media is custom made by BioSentinel. BoNT/A holotoxin was purchased from Metabiologics (Madison, WI, USA). Light-chain A (LC/A) was purchased from List Laboratories (Campbell, CA, USA). Antibodies against SV2A, B, and C were purchased from Synaptic Systems GmbH (Göttingen, Germany). Antibodies against GT1b and GD1a were purchased from MilliporeSigma (Burlington, MA, USA). Alexa Fluor 568 nm conjugated secondary antibodies were purchased from Thermo-Fisher. The heavy-chain receptor domain of BoNT/A (HcR/A) was produced by BioSentinel according to Baldwin et al. [48]. Tissue-culture flasks were purchased from Corning (Corning, NY, USA). Poly-D-lysine-coated 96-well plates were purchased from Greiner Bio-One (Kremsmünster, Austria) or Corning.

### 4.2. BoNT/A Sample Preparation

BoNT/A holotoxin (Metabiologics) was used for all characterization assays. For the semi-automated CBPA development and method qualifications, an internally generated BoNT/A reference (2 nM BoNT/A holotoxin, 2 mM lactose, 0.01% polysorbate-20, 0.05% HSA, 20 mM L-histidine, 100 mM NaCl) was used. Under- and over-potent samples of the BoNT/A drug product reference were generated by diluting the BoNT/A reference in BoSapient Assay Media (SAM; iBAM2 supplemented with 1x B-27, 2 mM GlutaMAX, and 18 mM NaCl). Where indicated, 100 U BOTOX^®^ and 200 U Xeomin^®^ vials were resuspended in SAM to the concentrations indicated.

### 4.3. Cell Growth and CBPA Procedures

Cells were continuously passaged every 3 or 4 days in growth media (RPMI 1640 with GlutaMAX supplemented with 10% fetal bovine serum, 0.1 mM non-essential amino acids, 10 mM HEPES, 1 mM sodium pyruvate, and 2 mM GlutaMAX) at 37 °C, 5% CO_2_ in T25, T75, or T225 tissue-culture flasks. During passaging, spent media was removed and the cell layer was rinsed once with 0.05% Trypsin/EDTA. Cells were then incubated with 0.05% Tryspin-EDTA for 5 min at 37 °C. Cells were collected and centrifuged before resuspending the pellet in fresh growth media. Cells were counter-stained with trypan blue and counted using a hemacytometer. Cells were then re-seeded into a new flask and/or an assay plate.

To perform the BoSapient CBPA, 4-day culture cells were plated into a poly-D-lysine-coated 96-well plate at a density of 50,000 cells/well in 100 µL of growth media and grown 19 ± 1 h at 37 °C, 5% CO_2_. The cells were then washed with 100 µL SAM before the addition of serially diluted BoNT/A toxin. The assay plate was then incubated for 48 ± 2 h at 37 °C, 5% CO_2_. Following incubation, the assay plate was washed three times with 100 µL/well phosphate-buffered saline (PBS) using an automated cell washer (Agilent BioTek, Santa Clara, CA, USA) leaving ~50 µL residual volume and immediately read on a fluorescence plate reader (Agilent BioTek) capturing YFP (ex ~500 nm, em ~540 nm) and CFP (ex ~440 nm, em ~485 nm) emissions. Where applicable, CBPA plates were imaged with an InCell Analyzer (GE Healthcare, Chicago, IL, USA) outfitted with bright-field, Texas Red, and YFP filter sets.

### 4.4. Semi-Automated BoSapient CBPA Execution

Reference and test BoNT/A samples in SAM were added to a deep-well staging plate and then placed in the Cybio FeliX automated liquid handling and pipetting platform (Analytik Jena, Jena, Germany) along with a reagent reservoir containing dilution and wash buffer (SAM), racks containing 1000 µL pipetting tips, 8- and 12-channel pipetting adaptors, and an additional head adaptor support. The Cybio FeliX was configured with a CHOICE pipetting head and connected to a dedicated computer running the Cybio Composer software(v2.66). Once the platform was loaded with all materials and reagents, each assay run was initiated by opening the custom method script developed and optimized by BioSentinel and selecting run. The automated pipetting platform then serially diluted the reference and test samples in the staging plate before pausing. The operator then added an assay plate (previously seeded with cells and incubated overnight as described above) and selected continue. The platform then washed the assay plate and applied the sample dilutions. The operator then removed the assay plate and placed it in an incubator (37 °C, 5% CO_2_) until the end of the assay.

### 4.5. Method Qualifications

The manual and semi-automated methods were qualified by executing 16 assay runs, yielding 30 total determined relative potencies with test samples at potencies ranging from 64 to 150% of a 4 pM nominal concentration (i.e., theoretical dilution) and generated from a 2 nM internal BoNT/A reference. To assess intermediate precision and transferability of the script to other CyBio FeliX systems, the qualification was run with two different pipetting heads (semi-automated method) or two operators (manual) over four independent testing days. A testing schedule and qualification acceptance criteria can be found in Supplementary Materials S1.

### 4.6. Data Analysis

JMP^®^, v17 (JMP Statistical Discovery LLC., Cary, NC, USA) and Prism, v10 (GraphPad Software, Boston, MA, USA) software was used for analysis. For any CBPA analysis, background emissions (from wells containing no cells) were subtracted from the YFP and CFP emissions for each well. Data are presented as emission ratios calculated by dividing the background-subtracted YFP emissions by the background-subtracted CFP emissions from a given well.

Relative potency calculations were performed using a custom JMP script developed by BioAssay Sciences and refined specifically for the BoSapient CBPA. The script imports the raw YFP and CFP emission data, background subtracts the emissions, determines the emission ratio for each well, and plots the dose–response curves (log 10 transformed emission ratio values versus log 10 transformed concentrations). Outlier analysis based on Jackknife Z (within group) and externally studentized residuals (between group) is used to identify outliers, and dose masking is performed to identify doses outside the linear range of the assay. Assay system suitability and validity evaluations include dose test, curve depth, parallelism, and linearity. The relative potency of each test sample is then determined relative to the reference using parallel line analysis (PLA) and an ANCOVA linear model.

Method qualification analysis for linearity, accuracy/bias, repeatability, intermediate precision, and range were completed with the aid of JMP using the equations described in Supplementary Materials S1.

### 4.7. Immunofluorescence Procedures

Cells were plated on poly-D-lysine-coated plates at 25,000 cells/well in 100 µL growth media and grown for 19 ± 1 h at 37 °C, 5% CO_2._ Growth media was then replaced with serum-free growth media. After 24 h, cells were fixed in 4% paraformaldehyde and 4% sucrose, and then permeabilized with 1.0% casein and 0.1% Triton X-100 in PBS. Cells were incubated with 4 µg/mL anti-SV2 antibodies specific to SV2A, SV2B, or SV2C in 0.33% casein and 0.1% Triton X-100 in PBS for 2 h at room temperature. Cells were washed three times with 100 µL PBS per well and then incubated with 4 µg/mL Alexa Fluor 568 nm conjugated secondary antibody in 0.33% casein and 0.1% Triton X-100 in PBS for 2 h at room temperature. The same procedure was followed for immunostaining with anti-GT1b and anti-GD1a antibodies. Representative images were captured using an InCell Analyzer equipped with bright-field, Texas Red, and YFP filter sets.

## Figures and Tables

**Table 1:** Relative potency results from CBPA runs at the indicated passage number and source vial position. For a given passage number and vial position, two BoNT/A test samples (TS1 and TS2) were subjected to the BoSapient CBPA and their potencies were determined relative to a reference sample of the same composition.

		Relative Potency (%)			
Passage Number	Vial Position	TS1	TS2	Mean	SD
6	middle	103.5	100.7	102.1	2.0
10	beginning	103.8	106.1		
	middle	105.5	99.8	103.5	2.3
	end	101.9	103.6		
16	beginning	104.3	104.7		
	middle	105.3	100.9	102.8	2.3
	end	99.6	102.1		
20	beginning	100.9	100.9		
	middle	100.9	103.5	103.1	3.5
	end	102.5	110.0		
26	beginning	103.1	103.2		
	middle	101.8	100.3	102.3	1.5
	end	101.1	104.4		
30	beginning	100.1	106.2		
	middle	100.3	100.9	100.5	3.1
	end	97.1	98.5		

**Table 2:** Summary of the manual and semi-automated BoSapient CBPA method qualifications.

Manual Method	All Data			Operator 1			Operator 2		
Theoretical Diution (% of Nominal)	Mean Determined Relative Potency (%)	SD	CV (%)	Mean Determined Relative Potency (%)	SD	CV (%)	Mean Determined Relative Potency (%)	SD	CV (%)
**64**	63.8	1.6	2.5	64.1	1.7	2.6	63.2	1.7	2.7
**80**	83.2	5.3	6.3	87.8	6.8	7.7	81.0	3.2	3.9
**100**	99.2	4.9	4.9	96.1	1.1	1.1	105.4	0.5	0.5
**125**	126.4	5.0	4.0	123.8	1.4	1.1	127.7	5.9	4.6
**150**	151.1	4.9	3.3	152.8	2.5	1.6	147.9	8.4	5.7
**Semi-Automated Method**	**All Data**			**Head 1**			**Head 2**		
**Theoretical Diution (% of Nominal)**	**Mean Determined Relative Potency (%)**	**SD**	**CV (%)**	**Mean Determined Relative Potency (%)**	**SD**	**CV (%)**	**Mean Determined Relative Potency (%)**	**SD**	**CV (%)**
**64**	65.0	1.9	2.9	65.9	1.4	2.2	63.1	1.3	2.0
**80**	81.5	1.9	2.4	81.3	0.7	0.9	81.6	2.4	3.0
**100**	100.5	2.3	2.3	100.5	2.5	2.5	100.7	2.7	2.7
**125**	124.2	2.2	1.8	126.3	0.8	0.6	123.2	1.9	1.6
**150**	146.6	4.9	3.3	147.7	4.3	2.9	144.4	7.1	4.9

**Table 3:** Accuracy and precision of the manual and semi-automated BoSapient CBPA methods.

Method	Theoretical/Expected Relative Potency (%)	Replicates	Between Operator/Head Variation (%)	Repeatability (%)	Repeatability (% of Tolerance)	Repeatability Acceptance	Intermediate Precision (%)	Intermediate Precision (% of Tolerance)	Intermediate Precision Acceptance
manual	64	6	0.40	1.38	15.8	pass	1.44	**16.4**	pass
manual	80	6	3.23	3.56	40.8	pass	4.81	55.0	pass
manual	100	6	4.37	0.78	9.0	pass	4.44	50.8	pass
manual	125	6	1.83	4.18	47.8	pass	4.56	52.2	pass
manual	150	6	2.31	3.85	44.1	pass	4.49	51.4	pass
manual	**mean**			**2.75**			**3.95**		
semi-auto.	64	6	1.31	1.14	13.0	pass	1.73	19.8	pass
semi-auto.	80	6	0.13	1.75	20.1	pass	1.76	**20.1**	pass
semi-auto.	100	6	0.12	2.10	24.1	pass	2.11	**24.1**	pass
semi-auto.	125	6	1.47	1.44	16.4	pass	2.06	**23.6**	pass
semi-auto.	150	6	1.56	4.17	47.7	pass	4.45	**50.9**	pass
semi-auto.	**mean**			**2.12**			**2.42**		

## Data Availability

The original contributions presented in this study are included in the article/Supplementary Materials. Further inquiries can be directed to the corresponding author.

## Supplementary Materials

The following supporting information can be downloaded at https://www.mdpi.com/article/10.3390/toxins18010045/s1, Supplementary Materials S1: Method qualification study design; Supplementary Materials S2: Validation of the automated script; Supplementary Materials S3: Variance testing of the semi-automated and manual methods; Supplementary Materials S4: Raw Data for Figure 2, Figure 3 and Figure 5; Table S1. Manual and Semi-automated Methods Qualification Data.

## Abbreviations

The following abbreviations are used in this manuscript:

4PL	4-parameters logistic
BoNT	botulinum neurotoxin
BoNT/A	botulinum neurotoxin serotype A
CBPA	cell-based potency assay
CFP	cyan fluorescent protein
FRET	Förster resonance energy transfer
HcR/A	heavy-chain receptor binding domain of BoNT/A
LC/A	light chain of BoNT/A
LSL/LL	lower suitability limit
MBPA	mouse-based potency assay
OOS	out of specification
PLA	parallel line analysis
PBS	phosphate-buffered saline
PPM	parts per million
RP	relative potency
SNAP-25	synaptosomal-associated protein, 25kDa
SV2	synaptic vesicle glycoprotein 2
USL/UL	upper suitability limit
YFP	yellow fluorescent protein
