# Supplementary material for: A Cell-Based Potency Assay for Determining the Relative Potency of Botulinum Neurotoxin A Preparations Using Manual and Semi-Automated Procedures
Source: Toxins (Basel). 2026 Jan 15;18(1):45. doi: 10.3390/toxins18010045 (PMC12845863; doi:10.3390/toxins18010045)
Supplement: Supplementary file 1 [file toxins-18-00045-s001.zip › Supplementary Materials S1.pdf]

S1 Appendix. Method qualification study design.

Study Design Evaluation and Power

The study is a main effects only design of experiments with main effects of Analyst (operator or head), and Day as the factors at each target theoretical relative potency. For a signal to noise ratio of 1.25 (difference in the signal/(RMSE/sqrt(n)), the power of the test will be 0.90 or greater. This indicates the study design is well powered to reliably detect differences in theoretical relative potency, day, and analyst.

Fig A. Power of Study Design

Power Analysis

Significance Level 0.05

Anticipated RMSE 1

Term	Anticipated Coefficient	Power
Intercept	1	0.999
Theoretical Relative Potency 1	1.25	0.906
Theoretical Relative Potency 2	1.25	0.906
Theoretical Relative Potency 3	1.25	0.906
Theoretical Relative Potency 4	1.25	0.906
Day	1	0.999
Analyst	1	0.999

Effect	Power
Theoretical Relative Potency	1

Method qualification study schedule. Method qualification was completed by executing 16 events or runs per method with day and operator (manual) or CHOICE pipette head (semi-automated) varied as indicated:

Table A. Qualification study schedule for manual and semi-automated methods.

			reference sample	test samples	
**event**	**day**	**operator or Choice head**	**reference (theoretical dilution)**	**test 1 (theoretical dilution)**	**test 2 (theoretical dilutio.)**
**1**	1	1	100	64	125
**2**	1	2	100	80	150
**3**	1	1	100	100	64
**4**	1	2	100	125	80
**5**	2	1	100	150	100
**6**	2	2	100	64	125
**7**	2	1	100	80	150
**8**	2	2	100	100	empty
**9**	3	1	100	64	125
**10**	3	2	100	80	150
**11**	3	1	100	100	64
**12**	3	2	100	125	80
**13**	4	1	100	150	100
**14**	4	2	100	64	125
**15**	4	1	100	80	150
**16**	4	2	100	100	empty

Theoretical dilutions of 64, 80, 100, 125, and 150% correspond to test samples with a starting concentration of 2.56, 3.2, 4, 5, and 6 pm, respectively.

Method qualification analysis and acceptance criteria. All analysis was completed in JMP version 17.1 software.

For accuracy/bias evaluation, the bias at each dilution was evaluated against the target theoretical % relative potency. If the bias as a percentage of tolerance was <=20%, then the assay was deemed accurate at the target theoretical dilution.

Upper Spec Limit = 125
Lower Spec Limit = 80
Mean = average (Constrained Relative Potency)
Bias = Mean RP – Theoretical RP for each dilution
Bias % Tolerance = ((abs(Bias))/(USL-LSL))*100
Bias Upper 95% CL = (Bias + -t Quantile(0.05 / 2, Number - 1)*(Col Std Dev(:RP, :Theoretical RP) / Root(number))) - :Theoretical RP

Bias Lower 95% CL = (Bias - -t Quantile(0.05 / 2, Number - 1)*(Col Std Dev(:RP, :Theoretical RP) / Root(Number))) - :Theoretical RP

For linearity evaluation, the theoretical dilutions of 64, 80, 100, 125, and 150% from the study design were used. A bivariate plot of the measured relative potency at all theoretical dilutions was constructed to demonstrate, visually, the linearity of the assay. The studentized residuals was measured from the linear curve. A quadratic fit of the studentized residuals was used to establish the linear range of the assay. Limits were set at ±1.96 of the studentized residuals.

The limits indicate the range where there is a 95% certainty the assay is linear. The intersection where the 95% confidence interval of the quadratic curve passes either linear limit was the end of the linear range of the assay.

For repeatability evaluation, the variance components of day and analyst were the main effects (inter-assay error) in the analysis and the within (intra) assay error was repeatability. Intermediate precision was calculated using the standard deviation of all assay results at each theoretical dilution. Partition of variation (POV) analysis was used to isolate the variance components. The variance components were converted to standard deviations and 95% CIs.

The following are the equations used for acceptance criteria for repeatability and intermediate precision:

Repeatability % of Tolerance = ((Repeatability/Root(n))*5.15) / (USL-LSL)*100
Repeatability % of Tolerance Acceptance Criterion<=60% of tolerance at each dilution
Repeatability Lower 95% CI= (Repeatability/Root(n)) * Root((n-1)/ChiSquare Quantile(1 - 0.05/2,Number-1))
Repeatability Upper 95% CI= (Repeatability/Root(n)) * Root((n-1)/ChiSquare Quantile(0.05/2, Number-1))
IP % of Tolerance = ((IP/Root(n))*5.15)/(USL-LSL)*100
IP % of Tolerance < = 70% tolerance at each dilution
IP Lower 95% CI= (IP/Root(n)) * Root((n-1)/ChiSquare Quantile(1 - 0.05/2, Number-1))
IP Upper 95% CI= (Intermediate Precision/Root(n)) * Root((n-1)/ChiSquare Quantile(0.05/2, Number-1))

In the equations above, ‘n’ refers to the number of independent determinations performed to obtain a reportable result (e.g., n=3). ‘Number’ refers to the number of determinations run during qualification for each theoretical dilution. The 5.15 multiplier for repeatability and intermediate precision covers long-term variation of the assay. 5.15 corresponds to covering 99% of the population.

The range of the bioassay assay was established by evaluating the results from each theoretical dilution for accuracy, repeatability, precision, and linearity. The range was based on the assessments explained above. The assay range was set based on the maximum range that meets all acceptance criteria:

Accuracy	≤20% for all theoretical dilutions
Repeatability	≤60% for all theoretical dilutions
Intermediate Precision	≤70% for all theoretical dilutions
Linearity	≤64% and ≥150%
