# Supplementary material for: A Cell-Based Potency Assay for Determining the Relative Potency of Botulinum Neurotoxin A Preparations Using Manual and Semi-Automated Procedures
Source: Toxins (Basel). 2026 Jan 15;18(1):45. doi: 10.3390/toxins18010045 (PMC12845863; doi:10.3390/toxins18010045)
Supplement: Supplementary file 1 [file toxins-18-00045-s001.zip › Supplementary Materials S2.pdf]

Supplementary Materials S2. Validation of the automated script.

Validation of the automated script - method. Once finalized, the automated pipetting platform script was validated for both observable and measurable events. A script validation protocol was written to validate the script that includes the protocols for observable and measurable events testing, acceptance criteria for each protocol, tables to record measurable events summary data, and a log of any deviations encountered during execution.

Observable events. After script development was completed and the script finalized, a Script Log was created that listed all script commands that result in any action on the part of the automated platform. These actions include turning on lights, user prompts or other interaction, switching pipetting adaptors, script pauses, movement from one position/well to the another, mounting and disposal of tips, and aspiration/dispensing parameters including volume, speed, and x-, y-, and z-positioning. The Script Log also indicates measurable events testing points described and used in the Measurable Events Log, described below.

A time-synced, multi-camera video of the automated pipetting platform system during script execution was recorded to allow observational verification of the Script Log. Cameras were positioned to capture all automated pipetting platform movements and actions as well as the run log displayed on the computer screen. After the video was recorded, the camera angles were edited together into a final video and stored on BioSentinel's server.

The video was then viewed, independently, by three BioSentinel scientists familiar with CBPA methods. Each scientist verified the movements according to the Script Log making note of any possible incorrect actions, omissions, or additional movements. The acceptance criteria for the observable events specifiy that all listed script actions in the Script Log, as observed by three scientists during script execution, contain no incorrect actions, omissions, or additional movements compared to the Script Log.

Measurable events. A Measurable Events Log was generated that describes the script steps and locations where execution should be paused for collecting dispensed or residual volumes for measurement and documentation. The Measurable Events Log also defines what script action (reset or continue) should be performed following collection of the removed volumes. For the assay plate, dispensed volumes were measured both in the context of the entire assay, where the dispensed volume adds to the previous residual volume, and in isolation, where the volume is dispensed and collected from a previously unused well.

Acceptance criteria were developed based on initial testing and the expected pipetting volumes defined within the script. Multiple instrument-independent sources of variation exist when measuring dispensed or residual volumes. These include volume losses resulting from sample transfer to the measurement tubes and variability when weighing the measurement tubes both before and after sample collection. Sample volume is more subject to be loss during transfer, so acceptance criteria of 20% below and 10% above the expected mean determined weight were established. Within a dispensing step, repeatability criteria were established so that no single volume was greater or less than 15% of the determined mean and <6% CV was set across all measured volumes. For residual volumes following aspiration, the measuring of small volumes is more prone

to pipetting inaccuracies due to the low volumes collected (<20 µl) and the adherence of liquid to surfaces. Furthermore, no residual volume was found to be ideal, so only an upper bound was specified. Acceptance criteria were the same for both Part 1, where data resulted from a continuously run method event, and Part 2, where data came from isolated pipetting events into the assay plate. The script measurable events acceptance criteria are listed below.

Script validation measurable events acceptance criteria.

Part 1

tube #	description	expected volume (ul)	mean determined weight (mg) [(Expected - 20%) - (Expected + 10%)]	lowest determined weight (mg)	highest determined weight (mg)	S.D.	%C.V.
1 to 12	staging plate - dilution media	200	180 - 210	>determined mean - 15%	<determined mean + 15%	NA	<6%
13 to 24	staging plate - samples	400	360 - 420	>determined mean - 15%	<determined mean + 15%	NA	<6%
25 to 30, 49 to 54	staging plate - final sample dilutions	400	360 - 420	>determined mean - 15%	<determined mean + 15%	NA	<6%
31 to 48, 55 to 72	staging plate - final sample dilutions	200	180 - 210	>determined mean - 15%	<determined mean + 15%	NA	<6%
73 to 84	aspirated assay plate	<10	<10	NA	<20	NA	NA
85 to 96	assay plate - wash addition	100	90 -120	>determined mean - 15%	<determined mean + 15%	NA	<6%
97 to 108	aspirated assay plate	<10	<10	NA	<20	NA	NA
109 to 120	assay plate - sample application	100	90 -120	>determined mean - 15%	<determined mean + 15%	NA	<6%

Part 2

tube #	description	expected volume (ul)	mean determined weight (mg) [(Expected - 20%) - (Expected + 10%)]	lowest determined weight (mg)	highest determined weight (mg)	S.D.	%C.V.
1 to 12	assay plate - wash addition	100	80 -110	>determined mean - 15%	<determined mean + 15%	NA	<6%
13 to 24	assay plate - sample application	100	80 -110	>determined mean - 15%	<determined mean + 15%	NA	<6%

Validation of the semi-automated script - results**.** The optimized script was validated based on verification of observable events and measurement of measurable events.

Observable events. Three operators independently watched a video of the automated pipetting platform executing the script and compared the execution of the script to a Script Log. No deviations between the Script Log and the automated pipetting platform actions were noted by any of the observers resulting in the script passing the acceptance criteria.

Measurable events. Measurable events testing was performed by a single operator in a 2-part study. Summary results from 2-part study are shown in tabular form below, green indicates the value meets the acceptance criteria.

Summary results for the measurable events testing. Numbers in green indicate that the value passes the acceptance criteria.

Part 1

Tube Number	Description	Expected Volume (µl)	Mean Determined Weight (mg)	Lowest Weight (mg)	Highest Weight (mg)	StDev	%CV
1 - 12	Staging plate- dilution media	200	192.45	185.6000	195.5000	2.7659	1.4372
13 - 24	Staging plate- samples	400	384.69	379.8000	389.0000	2.5819	0.6712
25 - 30, 49 - 54	Staging plate- final sample dilutions	400	391.27	384.5000	394.9000	2.8487	0.7281
31 - 48, 55 - 72	Staging plate- final sample dilutions	200	189.19	182.6000	199.3000	4.7981	2.5361
73 - 84	Aspirated assay plate	<10	7.13	1.4000	14.8000	5.0678	71.0437
85 - 96	Assay plate- wash addition	100.00	108.23	100.7000	116.8000	6.1290	5.6627
97 - 108	Aspirated assay plate	<10	7.88	3.2000	16.3000	4.2415	53.8038
109 - 120	Assay plate- sample application	100	104.01	102.3000	109.0000	1.7159	1.6498

Part 2

Tube Number	Description	Expected Volume (µl)	Mean Determined Weight (mg)	Lowest Weight (mg)	Highest Weight (mg)	StDev	%CV
1 - 12	Assay plate- wash addition	100	97.64	92.7000	100.3000	2.1690	2.2214
13 - 24	Assay plate- sample application	100	98.03	97.0000	98.9000	0.5610	0.5724

Individual sample variability within the dilutions plate are shown below. For each plot, the green dashed line indicates the expected volume while the red dashed lines denote the upper and lower bounds of the acceptance criteria. Measured values include the dispensing of diluent and stock dilutions and the volumes in each well following all dilution steps.

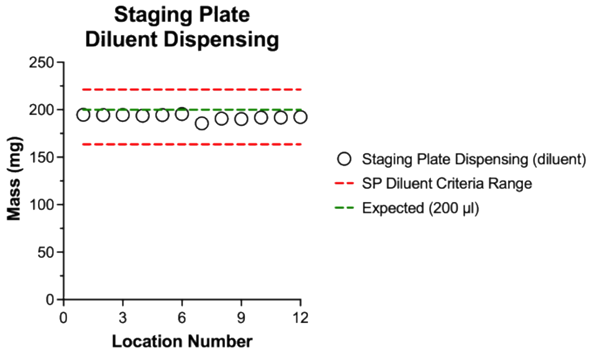

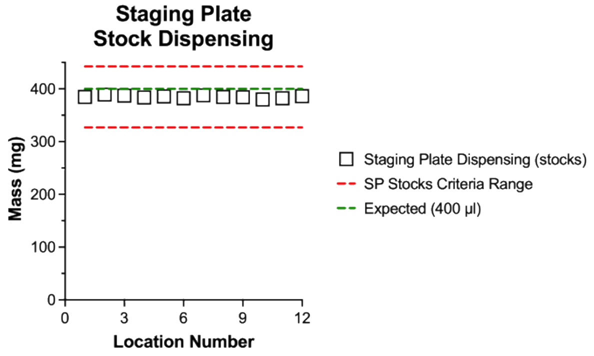

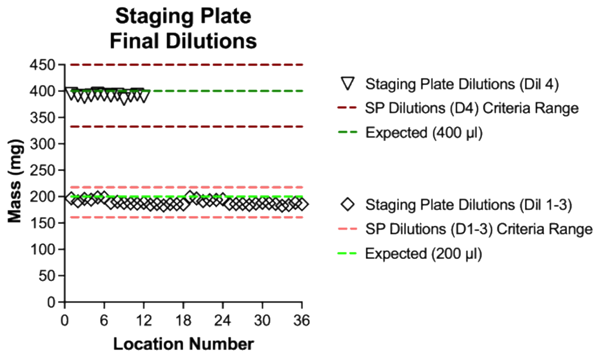

Staging plate measurable events testing during script validation. The semi-automated CBPA was run and sample volumes collected according to the validation protocol. In each plot, the green dashed line represents the expected volume based on the script and the red dashed lines indicate the upper and lower bounds of the acceptance criteria. Location number denotes the sample number of the tube collected for each volume at each measurement step. Mass is defined as the measured mass of the collected volumes obtained by subtracting the initial empty tube weight from the tube weight following volume addition. Staging plate diluent dispensing are samples of the dispensed diluent volume, staging plate stock dispensing are samples of the dispensed reference and test sample stocks, and staging plate final dilutions are all samples of the final reference and test sample dilutions 1 – 3 and dilution 4.

Measurements from the assay plate are shown below for both Part 1 (upper plots) and Part 2 (lower plots) testing. Measurements include residual volumes following buffer removal and dispensed volumes of both SAM wash buffer and final samples.

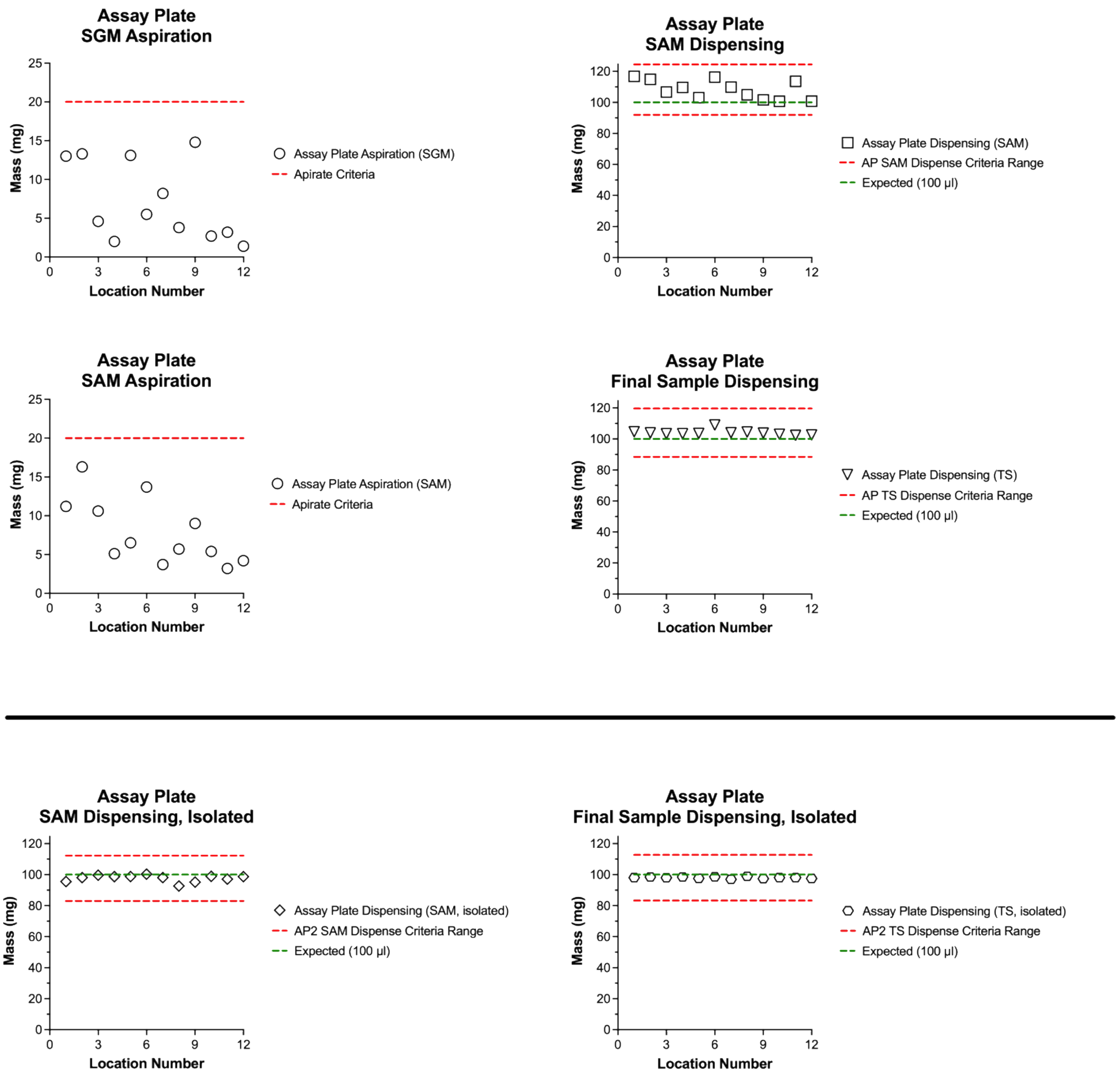

Assay plate measurable events testing from Parts 1 and 2 using the *Four Dose CBPA* script. Samples were collected and plotted from the assay plate similarly to Figure 4. The upper plots are from Part 1 testing, while the lower plots are from Part 2 testing. Assay plate SGM and SAM aspiration are samples of the residual volume after removal of the growth media (SGM) or assay media (SAM), assay plate SAM and final sample dispensing are samples of the dispensed SAM wash buffer or final assay dilutions.

The script passed all validation criteria for both observable and measurable events with no noted deviations.
