# Supplementary material for: A Cell-Based Potency Assay for Determining the Relative Potency of Botulinum Neurotoxin A Preparations Using Manual and Semi-Automated Procedures
Source: Toxins (Basel). 2026 Jan 15;18(1):45. doi: 10.3390/toxins18010045 (PMC12845863; doi:10.3390/toxins18010045)
Supplement: Supplementary file 1 [file toxins-18-00045-s001.zip › Supplementary Materials S3.pdf]

Supplementary Materials S3. Variance testing of the semi-automated and manual methods

The unequal variances test shows a reduction in variation when the semi-automated method is used compared to the manual method. One outlier was removed from the automation data following identification by Jacknife Z. Bias was used to eliminate the theoretical concentration effect. 95% confidence intervals for the semi-automated and manual methods' standard deviations do not overlap. Both methods exhibit 95% confidence intervals for bias that includes zero so neither method exhibits significant bias while the semi-automation method does exhibit significant improvements in repeatability.

Bias By Method

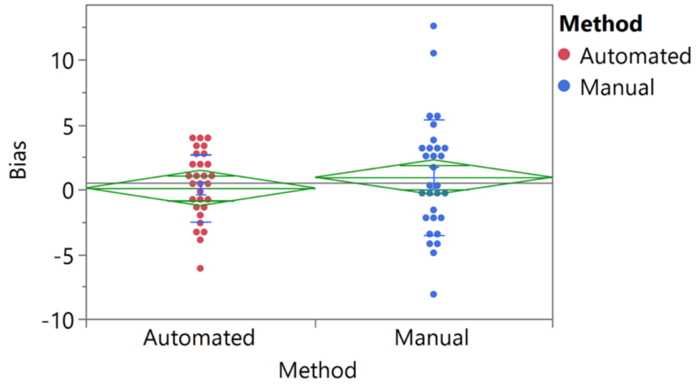

Means and Std Deviations

Level	Number	Mean	Std Dev	Bias Lower 95%	Bias Upper 95%	Std Dev Lower 95%	Std Dev Upper 95%
Automated	29	0.10	2.60	-0.88	1.09	2.06	3.51
Manual	30	0.93	4.45	-0.73	2.60	3.55	5.99

Tests that the Variances are Equal

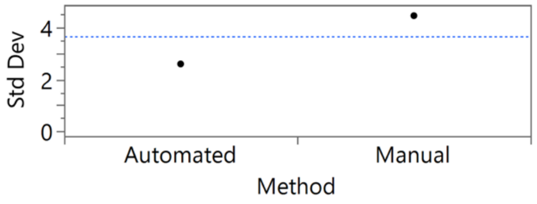

Level	Count	Std Dev	MeanAbsDif to Mean	MeanAbsDif to Median
Automated	29	2.60	2.120571	2.106897
Manual	30	4.45	3.475556	3.440000

Test	F Ratio	DFNum	DFDen	p-Value
O'Brien[.5]	4.4259	1	57	0.0398*
Brown-Forsythe	5.0013	1	57	0.0293*
Levene	5.6934	1	57	0.0204*
Bartlett	7.7522	1	.	0.0054*
F Test 2-sided	2.9451	29	28	0.0054*
