# Supplementary material for: A Cell-Based Potency Assay for Determining the Relative Potency of Botulinum Neurotoxin A Preparations Using Manual and Semi-Automated Procedures
Source: Toxins (Basel). 2026 Jan 15;18(1):45. doi: 10.3390/toxins18010045 (PMC12845863; doi:10.3390/toxins18010045)
Supplement: Supplementary file 1 [file toxins-18-00045-s001.zip › Supplementary Materials TableS1.pdf]

Table S1. Manual and Semi-automated Methods Qualification Data

Plate Reader File	Method	Analyst/Head	Theoretical Relative Potency	Determined Relative Potency
11212024 FeliX Hand Qualification Event 1.txt	Manual	1	64	66.4
11212024 FeliX Hand Qualification Event 1.txt	Manual	1	125	122.8
11212024 FeliX Hand Qualification Event 2.txt	Manual	2	80	76.3
11212024 FeliX Hand Qualification Event 2.txt	Manual	2	150	141.9
11212024 FeliX Hand Qualification Event 3.txt	Manual	1	100	95.8
11212024 FeliX Hand Qualification Event 3.txt	Manual	1	64	62.4
11212024 FeliX Hand Qualification Event 4.txt	Manual	2	125	128.3
11212024 FeliX Hand Qualification Event 4.txt	Manual	2	80	81.7
11222024 FeliX Hand Qualification Event 5.txt	Manual	1	150	149.6
11222024 FeliX Hand Qualification Event 5.txt	Manual	1	100	95.8
11222024 FeliX Hand Qualification Event 6.txt	Manual	2	64	64.4
11222024 FeliX Hand Qualification Event 6.txt	Manual	2	125	121.8
11222024 FeliX Hand Qualification Event 7.txt	Manual	1	80	83
11222024 FeliX Hand Qualification Event 7.txt	Manual	1	150	152.8
11222024 FeliX Hand Qualification Event 8.txt	Manual	2	100	105.7
12052024 FeliX Hand Qualification Event 9.txt	Manual	1	64	63.6
12052024 FeliX Hand Qualification Event 9.txt	Manual	1	125	124.8
12052024 FeliX Hand Qualification Event 10.txt	Manual	2	80	82.5
12052024 FeliX Hand Qualification Event 10.txt	Manual	2	150	153.8
12052024 FeliX Hand Qualification Event 11.txt	Manual	1	100	95.1
12052024 FeliX Hand Qualification Event 11.txt	Manual	1	64	63.8
12052024 FeliX Hand Qualification Event 12.txt	Manual	2	125	135.5
12052024 FeliX Hand Qualification Event 12.txt	Manual	2	80	83.3
12062024 FeliX Hand Qualification Event 13.txt	Manual	1	150	153
12062024 FeliX Hand Qualification Event 13.txt	Manual	1	100	97.6
12062024 FeliX Hand Qualification Event 14.txt	Manual	2	64	62
12062024 FeliX Hand Qualification Event 14.txt	Manual	2	125	125.1
12062024 FeliX Hand Qualification Event 15.txt	Manual	1	80	92.6
12062024 FeliX Hand Qualification Event 15.txt	Manual	1	150	155.6
12062024 FeliX Hand Qualification Event 16.txt	Manual	2	100	105
09272024 FeliX Qualification Event 1.txt	Automated	1	64	64.5
09272024 FeliX Qualification Event 1.txt	Automated	1	125	125.7
09272024 FeliX Qualification Event 2.txt	Automated	2	80	78.9
09272024 FeliX Qualification Event 2.txt	Automated	2	150	139.4
09272024 FeliX Qualification Event 3.txt	Automated	1	100	97.8
09272024 FeliX Qualification Event 3.txt	Automated	1	64	64.8
09272024 FeliX Qualification Event 4.txt	Automated	2	125	121.1
09272024 FeliX Qualification Event 4.txt	Automated	2	80	83.6
10032024 FeliX Qualification Event 6.txt	Automated	2	64	62.2
10032024 FeliX Qualification Event 6.txt	Automated	2	125	122.4
10032024 FeliX Qualification Event 7.txt	Automated	1	80	80.8
10032024 FeliX Qualification Event 7.txt	Automated	1	150	143.9
10032024 FeliX Qualification Event 8.txt	Automated	2	100	102.6
10042024 FeliX Qualification Event 9.txt	Automated	1	64	66.9
10042024 FeliX Qualification Event 9.txt	Automated	1	125	126.8
10042024 FeliX Qualification Event 10.txt	Automated	2	80	80.1
10042024 FeliX Qualification Event 10.txt	Automated	2	150	149.4
10042024 FeliX Qualification Event 11.txt	Automated	1	100	102.2
10042024 FeliX Qualification Event 11.txt	Automated	1	64	67.3
10042024 FeliX Qualification Event 12.txt	Automated	2	125	123.2
10042024 FeliX Qualification Event 12.txt	Automated	2	80	83.7
10102024 FeliX Qualification Event 13.txt	Automated	1	150	146.5
10102024 FeliX Qualification Event 13.txt	Automated	1	100	98.8
10102024 FeliX Qualification Event 14.txt	Automated	2	64	64
10102024 FeliX Qualification Event 14.txt	Automated	2	125	125.8
10102024 FeliX Qualification Event 16.txt	Automated	2	100	98.8
10172024 FeliX Qualification Event 15.txt	Automated	1	80	81.8
10172024 FeliX Qualification Event 15.txt	Automated	1	150	153.8
10252024 FeliX Qualification Event 5.txt	Automated	1	150	146.6
10252024 FeliX Qualification Event 5.txt	Automated	1	100	103
